# Integrated Chemometrics and Statistics to Drive Successful Proteomics Biomarker Discovery

**DOI:** 10.3390/proteomes6020020

**Published:** 2018-04-26

**Authors:** Anouk Suppers, Alain J. van Gool, Hans J. C. T. Wessels

**Affiliations:** Translational Metabolic Laboratory, Department of Laboratory Medicine, Radboud University Medical Center, Geert Grooteplein Zuid 10, 6525 GA Nijmegen, The Netherlands; anouk.suppers@radboudumc.nl (A.S.); alain.vangool@radboudumc.nl (A.J.v.G.)

**Keywords:** biomarker, clinical proteomics, chemometrics, statistics, preprocessing, classification models, feature reduction, review

## Abstract

Protein biomarkers are of great benefit for clinical research and applications, as they are powerful means for diagnosing, monitoring and treatment prediction of different diseases. Even though numerous biomarkers have been reported, the translation to clinical practice is still limited. This mainly due to: (i) incorrect biomarker selection, (ii) insufficient validation of potential biomarkers, and (iii) insufficient clinical use. In this review, we focus on the biomarker selection process and critically discuss the chemometrical and statistical decisions made in proteomics biomarker discovery to increase to selection of high value biomarkers. The characteristics of the data, the computational resources, the type of biomarker that is searched for and the validation strategy influence the decision making of the chemometrical and statistical methods and a decision made for one component directly influences the choice for another. Incorrect decisions could increase the false positive and negative rate of biomarkers which requires independent confirmation of outcome by other techniques and for comparison between different related studies. There are few guidelines for authors regarding data analysis documentation in peer reviewed journals, making it hard to reproduce successful data analysis strategies. Here we review multiple chemometrical and statistical methods for their value in proteomics-based biomarker discovery and propose to include key components in scientific documentation.

## 1. Introduction

In clinical research and clinical practice the biological state or condition of an individual can be determined by so-called molecular biomarkers, which are defined as detectible molecules in body fluids or tissues. Biomarkers have multiple applications depending on their intended use [1]: (i) diagnostic biomarkers detect diseases, (ii) prognostic biomarkers predict disease progression or recurrence, and (iii) predictive biomarkers predict treatment (medicinal or dietary) responses. Application of biomarkers is key to push personalized healthcare as they are individual, predictive, and preventive parameters [2]. Much biomarker research has been performed in the field of genomics, which resulted in biomarkers based on DNA and RNA levels. Nowadays, a shift towards proteomics biomarkers beyond protein expression is needed to properly assess protein function as reflected by post-translational modifications, alternative splicing, protein-protein interactions and protein turn-over rate [3]. Initially, proteomics research was performed to find one specific protein biomarker that by itself is able to characterize a disease. Multiple studies however show that for many diseases, such as cancer, this is not achievable due to interactions of complex cellular networks and the heterogeneous nature of these diseases [4,5,6,7]. Biomarker research therefore shifted towards discovery of biomarker panels that consist of multiple proteins.

**Figure 1:**
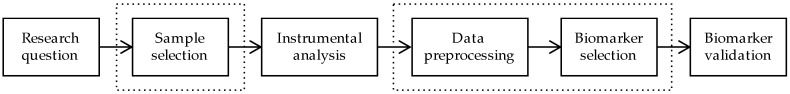
Biomarker discovery workflow. The encircled components highlight the focus of this review.

The research field of clinical proteomics aims to find such biomarkers by measuring thousands of peptide and protein levels in biological samples using tandem mass spectrometry (MS/MS). Data analysis techniques developed in the field of machine learning, chemometrics, data mining, and statistics are able to analyse and reduce large amount of data to identify biomarkers that are predictive for a biological state of an individual. Biomarker discovery has gained great interest within the field of clinical proteomics in the last decade for which a typical biomarker discovery workflow is depicted in Figure 1.

Even though developments have been made in hardware and data analysis techniques the translation of biomarkers to clinical practice is still limited. Too small sample sizes, poorly defined research questions, incorrectly justified statistical analysis, statistical overfitting, lack of instrumental standardization, and validation costs are several causes for this phenomenon [8,9,10]. Multiple reviews are available on how to address these individual challenges but do not discuss how choices made in one component of the biomarker discovery process influence the decisions for another component [1,4,11,12,13,14,15,16]. This review aims to discuss chemometrical and statistical aspects of the complete biomarker discovery process for clinical proteomics. Chemometrical and statistical choices need to be made across the complete biomarker discovery process and influence one another. Statistical calculations and chemometrical reasoning can be used to determine the optimal sample size in the experimental design stage of the project to ensure appropriate statistical power in the experiment. Pre-processing of acquired mass spectrometry data is performed using bioinformatics, statistical, and chemometrical methods to quantify and identify (poly)peptides and subsequently remove systematic biases, handle missing values, and reduce sample variability to yield ‘clean’ data ready for biomarker selection methods. Finally, machine learning and chemometrical approaches are used to select an optimal set of biomarkers that meet the defined prerequisites of the study. These key components in the proteomic biomarker discovery workflow, encircled in Figure 1, will be discussed and evaluated. Also, the interrelationship between choices made for every individual component will be examined and guidelines will be presented on how to select the most appropriate techniques for specific studies.

## 2. Sample Selection

### 2.1. Sample Size

Optimal sample size selection is a critical parameter in the experimental design of biomarker discovery studies. Ideally, a minimal number of samples should be used that suffice statistical requirements for biomarker identification in high dimensional proteomics data. The number of patients or healthy control donors should not be too large as this poses ethical, efficiency, and cost problems. Above all, the number of samples needs to be large enough to guarantee reliable statistical results with minimal false positives or false negatives rates. Ideally, one would like to select a priori the optimal sample size using calculations based on prior knowledge or statistical theory [17].

The established method to determine the optimal sample size in proteomics is the power calculation. A traditional power calculation is determined by the false positive rate (type I error, α), false negative rate (type II error, β) and the treatment effect size Δ. If *Z* is the percentile of a standard normal distribution the number of sample in each group is:

(1) $$n=\frac{2{\left(Z_{\frac{\alpha }{2}}+Z_{\beta }\right)}^{2}}{\Delta^{2}}.$$

The values for the false positive rate α and false negative rate *β* are typically selected as 0.05 and 0.20, respectively. These values are however fit for purpose and differ per biomarker discovery study. If the validation method to screen biomarkers candidates is efficient and able to screen many biomarker candidates simultaneously a higher false positive rate can be tolerated whereas the false negative rate should be minimized. If the validation method is cost demanding or low-throughput, only low false positive and false negative rates are accepted [12]. The treatment effect size Δ is based on the expected treatment difference divided by the standard deviation within groups, which is based on prior information [18]. In most biomarker discovery studies these parameters are typically unknown beforehand which poses a significant problem to justify parameter selection in power calculations and hence, the selected sample size. The sample size calculation based on the power calculation furthermore ignores the cost implication and ethical issues related to sample size selection [19].

Additional to these intrinsic issues two problems arise when power calculations are used for proteomics data with biomarker discovery as the end goal [20]. First of all, proteomics data is considered high-dimensional with a high level of correlation between data points which the power calculation does not take into account. Secondly, the power calculation aims to maximize the power of a test or model to separate between classes whereas classification algorithms used for biomarker selection in data analysis aim to maximize the prediction accuracy [21]. Different methods have been proposed that cope with high-dimensional data for sample size calculations but do not take the high level of correlation into account or are based solely on simulations [22,23,24,25]. Even though efforts have been made to translate the univariate power calculation to a multivariate classification purpose, there is still no method that overcomes all of these limitations.

Even if the optimal sample size could be determined correctly, the number of available patient samples might not be sufficient to avoid underpowered proteomics studies. Button et al. [26] stated that a small sample size undermines the reliability of the results but at the same time proposed multiple approaches on how to handle this problem. Most importantly, studies should always state that the experiment was underpowered irrespective of any approaches that were made to circumvent this problem. A potential solution to insufficient sample size is the option to form collaborative consortia in which groups of researchers combine data to increase the total sample size. However, it should be noted that even collaborative research does not solve the limited sample problem in case of rare diseases where only a few patients with a specific genetic or clinical phenotype are known worldwide. Furthermore, one should expect an increase of experimental variation that is introduced by decentralized sample collection and data acquisition at different research facilities.

Insufficient sample size in rare diseases raises the discussion whether or not such underpowered studies hold substantial value. Each independent study may inherently suffer from a significant number of false positive or negative results that could lead to misinterpreted biology or selection of putative biomarkers that fail clinical validation. On the other hand, underpowered studies may present the only available option to formulate hypotheses for pathogenic mechanisms in rare diseases. The data may also serve as independent additional evidence to help prioritize candidate biomarkers in the selection process of other related studies and may be used retrospectively in future studies to increase sample size as long as data is published according to FAIR data principles (Findable, Accessible, Interoperable, Reusable) [27].

### 2.2. Unbalanced Data

Limited sample size may also pose a specific problem for only one or some of the sample groups in clinical proteomics studies: in rare diseases the number of patients might be extremely limited but also healthy donor material might be scarce for a multitude of reasons. The sample cohort may therefore consist of what is called unbalanced or imbalanced data, in which e.g., the patient group is the minority case and the control group the majority case. Common classifier algorithms for biomarker discovery expect balanced class distributions [28]. When this is not the case the algorithm fails to represent the distributive character of the data which leads to samples of the minority class being classified in the majority class, decreasing the real classification performance.

There are multiple strategies to deal with the unbalanced data problem. One of the most common methods is to oversample the minority class or undersample the majority class [29]. These methods however increase computation time as the classification model needs to be performed multiple times, and with oversampling the same data is re-used multiple times which can create a bias. There are classification algorithms who inherently solve the problem of imbalanced data by for example adding a so called cost function [28]. One should therefore always study the mathematical background of an algorithm to determine if unbalanced data poses a problem for the classification algorithm.

## 3. Data Preprocessing

Many bioinformatics tools are available for the analysis of mass spectrometry data [30,31,32,33] and can roughly be divided into two categories. The first category of software exclusively quantifies peptides and proteins that were identified via MS/MS database searches prior to any statistical analysis to identify differential (poly)peptides. This is in contrast to the second category of software that quantifies yet unannotated LC-MS signals first from which differential features are detected that are subsequently annotated by MS/MS database search information [11]. The most common workflow is to first identify the peptides and subsequently quantify the LC-MS signals of these peptides [34]. This ensures that the identity of biomarkers is known after statistical analysis. It is, however, important to realize that peptides not identified by MS/MS are not quantified which means that key biomarkers might be overlooked [35]. The second workflow first quantifies LC-MS feature data, which is directly analysed to identify differential features before MS/MS identification results are mapped to the quantified features. With this method all possible differential features are taken into account with the potential outcome that a biomarker is not identified. However, additional targeted MS/MS analyses can be performed to confirm the identity of the biomarker with mass spectrometer settings that favour MS/MS quality over quantity. Both workflows ultimately lead to a quantitative feature matrix, in which the rows and the columns correspond to extracted features and samples. The features are characterized by *m*/*z*, charge, and retention time and represent an identified peptide/protein.

The resulting quantitative feature matrix needs to be pre-processed before statistical analysis, which can consist of normalization, missing value imputation, and pre-treatment methods such as centering, scaling, or transformations.

### 3.1. Normalization and Missing Value Imputation

Due to small variations in the experimental conditions systematic biases of non-biological (experimental) original can occur. The exact reason of the bias might be unknown and may not be solved by adjusting experimental settings. To eliminate this bias normalization is applied on the quantitative feature matrix to allow equitable comparisons between samples. Normalization consists of two distinguishable components: the mathematical function for normalization, and the feature selection approach used to select features that are used by the mathematical function. Webb-Robertson et al. [36] discusses the feature selection approaches and Valikangas et al. [37] explains the different mathematical functions for normalization. Most mathematical functions for normalization originate from the field of DNA microarray technology and can be divided into two categories based on whether or not the bias is dependent on the signal intensity. Both papers not only explain but also evaluate mentioned normalization techniques and conclude that there is not one specific normalization method that works best on all datasets due to the different nature of systematic biases. They therefore argue to apply multiple normalization techniques and systematically evaluate which normalization method is best able to eliminate the bias of the dataset. One can for example measure a control sample multiple times and select the normalization method that results in the least amount of variability between the measurements.

Substantial missing feature intensities are typical for holistic LC-MS/MS datasets. This causes a problem as most statistical algorithms require a complete data matrix with no missing values [38]. Missing values can be caused by technical or biological reasons: the peptide could be present but the intensity was below the instrumental detection limit or the peptide could only be present in some of the samples [39]. There are several ways to deal with missing values [38]: (i) remove the feature if one of the samples contains a missing value for that feature, (ii) employ statistical methods which can handle missing values, or (iii) use statistical models that impute missing values. For clinical proteomics the first option is not preferred due to the fact that peptides or proteins might be exclusively expressed in healthy or disease conditions. Removal of features with missing values would lead to a dramatic loss of information and effectively excludes black or white biomarkers from detection. Alternatively, one could apply a group count missing value approach. If for a feature, values are missing for more than a predefined percentage of the samples in either sample group, that feature could be removed. The missing values can also be imputed to obtain a complete data matrix. Lazar et al. [40] and Webb-Robertson et al. [38] review and evaluated statistical methods for imputing missing values and both argue that not one imputation strategy is generally advantageous in any situation. Nevertheless, missing values should be addressed by group count filtering and/or missing value imputation prior to subsequent statistical analysis. It is recommended to evaluate different strategies on a subset of the data to select the optimal approach based on the performance of the statistical analysis that will be applied.

There is no consensus for the order of which intensity normalization and missing value imputation should be performed. The fact that many normalization methods require a matrix with no missing values gives the indication that missing value imputation should be performed prior to normalization. This could however obscure the bias that normalization techniques should remove. This effect would be larger with an increasing number of missing values. Karpievitch et al. [39] therefore proposed to first perform normalization prior to missing value imputation.

### 3.2. Pre-Treatment Methods

**Figure 2:**
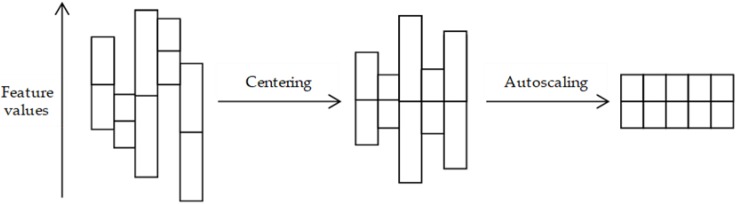
Graphical representation of the pre-treatment effects by data centering and autoscaling methods. This figure represents five samples, for which each of the vertical boxes is the feature value distribution of one sample, with the mean depicted as a horizontal bar inside the box. Centering removes the offset from the data so that the sample means become zero and autoscaling converts the data so that the standard deviation becomes one.

Samples each have a different degree of variability that could influence the biomarker discovery process as statistical methods compare the variability between and not within samples. The pre-treatment methods centering, scaling, and transformation minimize the sample variability so that this variability does not influence the data statistics [41]. Centering removes the offset from the data to adjust for the difference between low and high abundant peptides/proteins to shift the focus of the analysis towards the variation between samples. This is achieved by converting the mean of a sample to zero, so that the value of a feature from a sample becomes:

(2) $${\tilde{x}}_{ij}=x_{ij}-{\bar{x}}_{i},$$

where $x_{ij}$ is the original feature value and ${\bar{x}}_{i}$ the mean of all features from that sample. The common scaling method autoscaling performs centering and changes the feature value of a sample so that the standard deviation of that sample becomes one, which adjusts for the differences in fold changes between peptides/proteins:

(3) $${\tilde{x}}_{ij}=\frac{x_{ij}-{\bar{x}}_{i}}{s_{i}},$$

where $s_{i}$ is the standard deviation of all features from that sample. Both methods are illustrated in Figure 2.

A well-known transformation method is log transformation which converts the feature values to a more uniformly spread distribution, which allows for the application of parametric tests when the distribution of the data is skewed to the right:

(4) $${\tilde{x}}_{ij}=10\log\left(x_{ij}\right)$$

Selection of the appropriate pre-treatment method depends on the properties of the dataset since the amount and type of variability differs between samples. A generally accepted method for evaluation of pre-treatment methods is to perform Principal Component Analysis (PCA) [42]. A PCA scores plot of the samples is able to show the variance within and between the samples of each group. The best performing pre-treatment method should show a PCA scores plot with the smallest within group spread and largest between group distance. A detailed explanation of the PCA algorithm will be discussed in Section 4.1 of this work, and Van den Berg et al. [41] gives a detailed explanation on how to use PCA to evaluate different pre-treatment methods.

The specific order to execute the pre-treatment method depends on the type of statistical analysis. If biomarker discovery is based on univariate statistics, such as a *t*-statistic, the pre-treatment method needs to be carried out globally on the complete data matrix before statistical analysis. If a multivariate or machine learning technique is chosen for which the data is separated into a training and validation set, such as Partial Least-Squares Discriminant Analysis, centering and scaling needs to be performed individually on the training and validation sets to ensure independency [43]. There are furthermore types of statistical analysis that are not influenced by scaling methods. Tree-based algorithms, such as Random Forest, are not affected by transformations of the features [44]. In these algorithms a tree is built on the basis of decision rules. At each node of the tree values of a feature are compared and a threshold value is determined which is able to separate the groups.

## 4. Biomarker Selection

The primary goal of data analysis in biomarker discovery studies is to identify features that are able to correctly classify the samples in two or more groups, e.g., healthy vs diseased or different disease states. Feature selection methods are not only applied to retrieve biologically meaningful biomarkers but are also used to reduce the number of features required to discriminate between sample groups [45]. This dimensionality reduction is an important step in the data analysis process due to the fact that proteomics datasets typically suffer from the small-n-large-p problem; the number of features is far greater than the number of samples. Reducing the number of features avoids the risk of overfitting, thereby improving classification accuracy, lowering the computational costs and maximizing the chance of subsequent biomarker validation.

Feature reduction is typically performed in the data analysis step, but dimensionality reduction can already be accomplished during sample preparation/data acquisition or in the data pre-processing procedure. Alternatively, prior knowledge or pilot experiments can be used to define a list of putative biomarker candidates that can be studied in a targeted fashion. This removes the need to perform a holistic study that would only increase the number of non-relevant features and thereby increase dimensionality, which influences the prediction accuracy. As discussed in the pre-processing section, removal of features with missing values or applying a group count missing value approach during data pre-processing already lowers the number of features and reduces dimensionality prior to data analysis.

**Figure 3:**
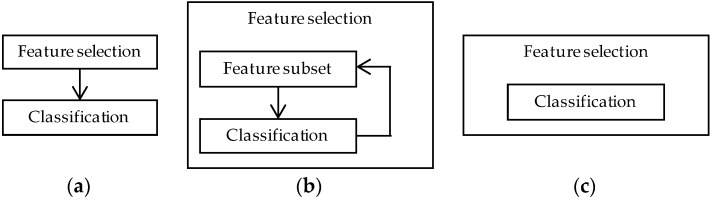
(**a**) Filter, (**b**) wrapper, and (**c**) embedded feature selection methods. Filter methods perform the feature selection independently of construction of the classification model. Wrapper methods iteratively select or eliminate a set of features using the prediction accuracy of the classification model. In embedded methods the feature selection is an integral part of the classification model.

Feature selection methods reduce the number of features by eliminating features that present redundant information or selecting relevant features. The feature reduction methods can be divided by how they are coupled to the classification or learning algorithms, depicted in Figure 3 [46]. A filter method reduces the number of features independently of the classification model. Wrapper methods wrap the feature selection around the classification model and use the prediction accuracy of the model to iteratively select or eliminate a set of features. In embedded methods the feature selection process is an integral part of the classification model. Before detailed discussion of the different feature selection methods, a selection of the most common classification and learning algorithms will be reviewed.

### 4.1. Classification Methods

#### 4.1.1. Principal Component Analysis

Principal Component Analysis (PCA) seeks an orthogonal transformation of the features that best explain the variance in the data [42]. The resulting transformation is described by uncorrelated variables called principal components, which are ordered according to the amount of variance they explain. The first principal component describes the largest variability in the data as possible followed by succeeding principal components which account for the highest variance possible orthogonal to the variability of the previous components. The results of PCA are described as scores and loadings, the scores contain the transformed data per principal component and the loadings contain the weights for each original feature per principal component. PCA is not a classification technique but due to its ability to describe the variability in the data by a handful of principal components it is widely used for feature reduction. PCA is also a commonly used method for exploratory analysis where a scores plot shows the underlying structure of the data by plotting the first principal component versus the second principal component.

#### 4.1.2. Partial Least Squares Discriminant Analysis

A classification variation of PCA is Partial Least Squares Discriminant Analysis (PLS-DA) [47]. This method constructs a linear multivariate model by transformation of the data which maximizes the covariance between the dataset and the group labels that need to be predicted. The transformed features are called latent variables that are described as scores in a similar fashion to PCA. A measure for the individual feature importance is provided by the loadings weights, regression coefficients, or variable importance in projection (VIP) [48]. Classification of test samples are determined by applying the regression coefficients to the sample features. It is common to show the result of the PLS-DA classification by a scores plot by plotting the scores of the first latent variable versus the second latent variable. This does however give an overrepresentation of the classification as correlations can be present by chance and a PLS-DA scores plot will this enlarge correlation [49].

#### 4.1.3. Support Vector Machines

Support Vector Machines (SVM) is a classification algorithm which searches for the hyperplane that separates two groups with the greatest distance [50]. This hyperplane is achieved by a small subset of the samples called support vectors. Test samples are classified according to which side of the hyperplane they end up. For linear cases SVM produces a weight vector corresponding to the feature importance. For nonlinear cases the so-called ‘kernel trick’ is applied where the data is transformed using for example a polynomial, radial basis function or sigmoid function to facilitate the search for a hyperplane. When using a kernel function the SVM algorithm does not produce a measure for feature importance. SVM for non-linear cases can therefore only be used in combination with a wrapper feature selection method [44].

#### 4.1.4. Random Forest

The Random Forest (RF) algorithm is a classification algorithm belonging to the family of decision trees [51]. The RF model is constructed by building an ensemble of many decision trees. Every tree is generated using a different set of bootstrap selected samples called in-bag samples. At every split in the decision tree a random subset of features is used. The importance of each feature is determined by the decrease in classification margin if the values of that feature are permuted across the out-of-bag samples. Test samples are classified by determining the number of votes per group label over all trees in the model.

#### 4.1.5. Artificial Neural Networks

Artificial Neural Networks (ANN) is a deep learning algorithm inspired by biological neural networks [52]. An ANN consists of an input layer, multiple hidden layers, and an output layer inspired by biological nervous systems that are interconnected via nodes. ANN requires training data and a desired output, e.g., correct classification of the group labels. The algorithm is self-learning and therefore requires no mathematical function as input. Each layer in the network transforms the training data and passes it on to the next layer, increasing the complexity and detail of the learning process until the desired output is reached. Test samples are presented to the learned network which classifies the samples. A measure of feature importance is given by a weight vector.

### 4.2. Feature Selection Methods

#### 4.2.1. Filter Methods

Filter methods select features on the basis of a calculated score by looking only at the intrinsic properties of the data. The calculated scores are used to remove low-scoring features and retain high-scoring features. Classical filter techniques are the t-statistic and its multiclass variant ANOVA, which allows for a comparison of more than two groups. The *p*-values calculated for every feature are the scores by which features are removed or retained. The selected features are subsequently used to build the classification model to find discriminating biomarkers of the remaining features [53]. A common disadvantage of these techniques is their univariate nature. The feature scores are calculated for every feature individually and interactions between features are ignored, which can lead to a decrease in classification performance as disease effects can result from a combination of features.

A multivariate feature selection method is PCA which transforms the features to principal components that are subsequently used to build the classification model. Selection of the number of principal components is a critical step, selecting too many components can introduce noise while selecting too few may lead to discarding valuable information. Cangelosi and Goriely review the most common methods for selecting the optimal number of principal components [54]. They recommend looking for a ‘consensus dimension’ given by multiple stopping techniques. The advantage of using all initial features to construct principal components comes at the cost of comprehensibility, the components are often not straightforward to interpret as they will be the orthogonal transformations of the original features.

Because the filter method needs to be performed only once prior to building the classification model, the techniques are fast and scalable. The filter methods do however require a parameter which specifies the cut-off value of the scores calculated for the features. Section 4.3 discusses this point in more detail.

#### 4.2.2. Wrapper Methods

In the wrapper approach the prediction accuracy of a classification model is used to determine the optimal feature subset. Different possible feature subsets are defined and their performance is evaluated by a classification algorithm. The classification model is first constructed using a subset of samples called the training set after which the model is evaluated by the remainder of the samples called the test set. The performance is measured in terms of prediction or classification accuracy. With the increasing number of features in proteomics data the number of subsets that needs to be evaluated increases dramatically. As a consequence, algorithms typically do not evaluate all possible feature subsets but use heuristic search methods and ‘wrap’ around the classification model to search for the optimal feature subset. Due to the nature of the search algorithm the wrapper methods are multivariate and take interactions between features into account. The dependency of the feature selection method on the classifier model performance can be an advantage or disadvantage. The selected features that result from the wrapper methods are features with good classification power but if the prediction accuracy of the model is low one cannot be certain about the selected features. Apart from the dependency on the classifier, wrapper methods can be computationally intensive when the classifier has a high computation cost and can be prone to overfitting.

Two common wrapper methods are Recursive Feature Elimination (RFE) [55] and Genetic Algorithm (GA) [56]. RFE can be combined with all classification models but is commonly coupled with SVM. In the RFE approach all features are first used to train the SVM classifier from which the calculated weight vector is used to remove features with the lowest weight vector value. The SVM classifier is then trained on the remainder of the features and the process is repeated until an optimal subset is established.

The Genetic Algorithm (GA) is a wrapper method that is based on a natural selection process that mimics biological evolution. A population is defined by individuals in which every individual contains a different feature subset. In each iteration of the algorithm, called generation, a fitness value is evaluated for every individual in the population. The fitness value is a parameter of choice, which is the prediction or classification accuracy of the classifier when GA is used for feature selection. The next generation is subsequently formed by modifying the population using mutation, crossover, and selection operations based on the fitness value ranking of the individuals. Over successive generations, the population evolves towards the optimal solution that shows the lowest fitness value. When the optimal solution is reached, the individual with the lowest fitness value is selected as optimal feature subset. In principal all classification models can be used to determine the fitness value of genetic algorithms. In practice Support Vector Machines (SVM) [57], k-Nearest Neighbours (k-NN) [58], and Random Forests (RF) [59] have been used in proteomics studies. All three classification algorithms are powerful classification methods and have low computation costs, which is a requirement for classifier used in wrapper methods.

#### 4.2.3. Embedded Methods

In embedded methods the feature selection is based on a score calculated by the classification model. A classification model is constructed using a training set and the prediction accuracy evaluated with a test set. The classification model that was build gives the performance value for every feature. Low scoring features will be removed and high scoring features retained, but different than filter methods the performance measure of the features is calculated by the classification model. Because the model is built only once to determine the feature scores, embedded methods have far less computational costs compared to wrapper methods, which require the construction of multiple models due to their iterative process. The embedded methods do however require a parameter which specifies the cut-off value of the scores calculated for the features. Section 4.3 discusses this point in more detail. Common methods are the VIP value for PLS-DA [60] and the weights of ANN input features [61].

### 4.3. Parameter Selection

**Figure 4:**
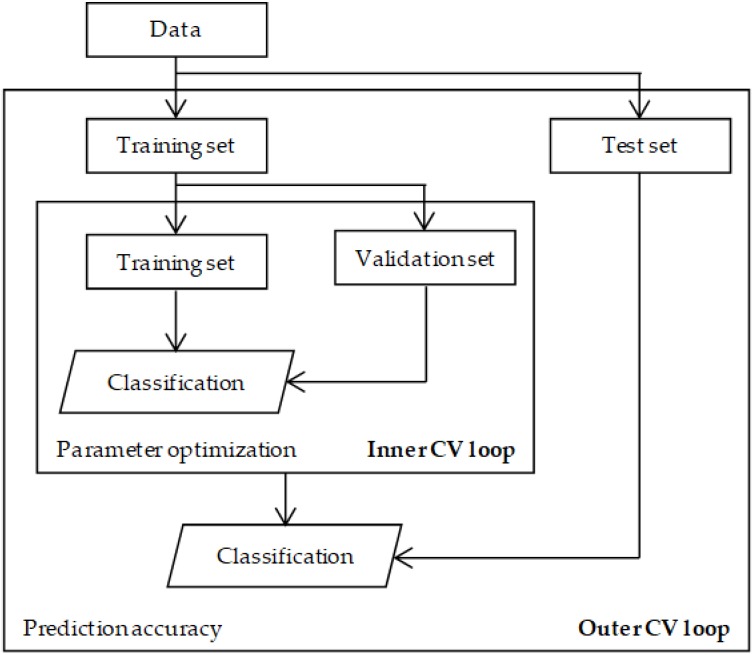
Schematic overview of a double cross-validation procedure. The samples are split into a training and test set to evaluate the prediction accuracy in the outer cross-validation (CV) loop. The training set is subsequently split into a training and validation set to optimize the parameter specific for a classifier in the inner cross-validation loop.

Both the classifier and feature selection methods require parameter values to be selected which have a significant impact on the final outcome of the analysis. These include the number of principal components, latent variables, the kernel method, and the number of trees for PCA, PLS-DA, SVM, and RF respectively. For every classification model this parameter can be optimized using a double cross-validation (2CV) procedure depicted in Figure 4. As described for wrapper methods, the samples are first split in a training set and a test set to construct and evaluated the model based on prediction accuracy. This cross-validation is called the outer loop. In the double cross-validation scheme the samples in the training set are again split into a training and validation set to select the optimal value of the parameter, which is called the inner loop. This double cross-validation ensures that there is no dependency between the samples used for parameter optimization and prediction error calculation. Westerhuis et al. [43] provides detailed information and an example using PLS-DA on how to construct a double cross-validation procedure.

Additional to parameter optimization for the classification algorithm, the wrapper methods require the selection of the number of features for the feature subsets. For the wrapper methods there is no rule of thumb for the selection of the number of features but the computational resources are the biggest determining factor for this. If the number of features in a subset is small more combinations of feature subsets are possible which increases the number of classification models that need to be build. Every classification model that is built requires computation time, so the smaller the feature subset the larger the computation cost.

The filter and embedded methods require a parameter which specifies the cut-off value of the scores calculated for the features. The selection of the cut-off value depends on the algorithm for which the scores are calculated. There are methods that have a common cut-off value, such as the 0.05 cut-off point for the univariate t-statistic and ANOVA methods. For some techniques the cut-off value is dependent on the constructed classification model and ranges around a preferred cut-off value, the value of 1 for the PLS-DA VIP score for example [62]. Not only the cut-off value but also the number and type of features that are selected are important and depend on the type of research and result that is required. When the final set of selected features is to be validated by high-throughput follow-up experiments there is no need to be conservative. In such cases, it might be more important to avoid false negatives rather than false positives. On the other hand, when only a limited number biomarker candidates can be validated in follow-up experiments, it is important to avoid false positives at the expense of false negative results.

### 4.4. Evaluation and Validation

The performance of a feature selection method is evaluated and validated based on the prediction accuracy of the classifier, and the statistical significance and stability of the selected features. Because univariate methods are not based on classification algorithms the performance is determined differently from multivariate methods.

A univariate test is deemed significant if the calculated *p*-value is lower than the α-level, the significance level which is often set to 0.05. However, using univariate methods for feature selection in proteomics data inherently leads to the so called multiple testing problem [63]. For feature selection numerous univariate tests are performed in a single experiment which increases the chance of finding false positives. The solution to the multiple testing problem is to adjust the α-level to maintain an acceptable false-discovery rate (FDR); the probability that a test produces a false positive result. Two common methods for controlling the number of false positives when performing multiple tests are the Bonferroni correction and the Benjamini-Hochberg correction [64]. The Bonferroni correction [65] changes the α-level at which a test, and therefore features are declared significant. If *m* tests are performed the level at which a test/feature is presumed to be significant becomes α = 0.05/*m*. This correction however is known to be conservative, especially in proteomics studies where the number of features are high. The α-level becomes so small that only a handful of features are deemed significant and the number of false negatives increases. A less conservative method is the Benjamini-Hochberg correction [66]. The *p*-values are ranked from low to high and are recalculated using α * (*i*/*m*), with *i* representing the rank position. The tests/features with a recalculated value lower than the α-level are declared significant. The choice of the preferred method depends on the FDR that is accepted in the biomarker validation process, as discussed in Section 4.3. The statistical significance of a *p*-value can additionally be determined by resampling techniques which is discussed at the end of this section.

Multivariate classification methods are evaluated with a performance measure and a corresponding significance value that are determined independently. The performance measures are based on how well the classification model is able to correctly classify a sample from the test set to its respective class. The sample can then be categorized as a true positive, true negative, false positive or false negative and stored in a confusion matrix. For binary classification the confusion matrix is illustrated in Table 1, for multiclass classification a confusion matrix is derived for every combination of classes.

With the confusion matrix the most common performance measures can be derived that are listed in Table 2. For multiclass cases the performance measures can be macro-averaged where the overall performance measure is the average of the performance measure for every class combination or micro-averaged where the overall performance measure is calculated by an overall confusion matrix which is the sum of all confusion matrices for every class combination [67].

Every performance measure has a different focus: NMC focuses on misclassifications, accuracy on the overall effectiveness of the classifier, sensitivity and specificity on correctly classifying positives and negatives respectively, and the AUC on the ability to avoid false classification. These differences make it difficult to compare performance measures between different classification methods as different performance measures could advocate distinct methods. It is, therefore, advised to not only report the final performance measure but also document the confusion matrices to improve transparency of results.

To determine significance of the performance measures and therefore stability of the classification model resampling techniques can be used. Common resampling techniques are bootstrapping, jackknifing, or permutation tests, of which the latter is typically used. Permutation tests evaluate if the performance measure is significantly better compared to any other random classification [68]. First the class/group labels are randomly permuted over the samples. The feature selection model that has been performed on the original data is performed again on the permuted data with random class/group labels. This procedure is repeated multiple times forming a distribution for performance measures of the random data which is not expected to be significant, a H0 distribution. The performance measure is said to be significant if the original (not permuted) data is outside the 95% or 99% confidence intervals of the H0 distribution.

### 4.5. Which Method to Choose?

Although most commonly used, the feature selection and classifier methods mentioned in the previous sections are only few of the many algorithms available. Even though multiple studies evaluated these feature selection methods there is not one method that outperforms all other methods in these studies [44,69,70]. The selection of the most suitable method is determined by the properties of the dataset, computational resources, the type of biomarker that is searched for and the validation process available after feature selection.

The number of sample groups in the dataset already gives a preference to certain classifiers. When one disease group is compared to healthy controls the classification problem is called binary for which all univariate and multivariate methods can be used. The number of applicable classifier algorithms however decreases when three or more groups are compared, typically referred to as multiclass classification. The basic PLS-DA and SVM algorithms do not support multiclass classification. Extensions have been proposed for these methods but require additional parameters that increase model complexity [49,71]. RF and ANN on the other hand are intrinsically capable of classifying both binary and multiclass problems.

The computation time needed to perform a feature selection procedure is an important decision factor that depends on the method of choice. Filter methods are fast and scalable, whereas wrapper methods have high computational costs. Additionally, the type of classifier and how the classifier is used has an influence on the computation time. RF is a fast algorithm when applied exclusively for classification purposes but demands high computation power if used for feature importance calculations. In addition, the number of parameters that need to be optimized significantly increases computation time. This means that an increasing number of pilot calculations on subsets of the data need to be performed to determine optimal parameters settings.

The choice of univariate or multivariate methods depends on the type of biomarker that is searched for. If the biomarkers of interest are single markers that by themselves can be used to classify samples from each group, univariate methods are the method of choice. Multivariate methods are preferred if the sample classification is expected to be defined by a set of biomarkers that are interrelated. If this is not known a priori it is advised to apply both univariate and multivariate methods as they are able to extract complementary information [72].

The experimental validation stage for biomarker candidates that will be performed after feature selection needs to be taken into account on how to execute the preferred methods with respect to false positive and negative rates that can be tolerated as discussed throughout this review.

## 5. Conclusions

Here, we provide a comprehensive overview of the individual chemometrical and statistical steps in the context of the full biomarker discovery workflow. Key decisions have to be made prior to starting proteomics data analysis that depend on the characteristics of the data, the computational resources, the type of biomarker that is desired and the subsequent biomarker validation strategy. All steps are interrelated and decisions made for one component directly influence the decisions for another. This review aims to provide the theoretical concepts behind the individual steps but also to guide researchers in how to apply these methods for the discovery of pivotal biomarkers.

All the decisions made in the biomarker discovery process ultimately determine which biomarkers are selected from high dimensional data with a specific biomarker application in mind. It is, therefore, crucial to thoroughly document all steps and related parameters of the full workflow to maximize the applicability of results for subsequent validation studies. Moreover, it is also required to enable peer scientists to evaluate and replicate study results. We therefore make the following recommendations when publishing proteomics biomarker discovery research: (i) all chemometrical and statistical decisions made in the complete biomarker discovery procedure should be thoroughly documented and substantiated, (ii) raw, not only (pre)processed data, should be made available, (iii) confusion matrices used for calculation of the performance measures for the feature selection methods should be supplied, and (iv) scripts used for the data analysis should be made available. The Minimum Information about a Proteomics Experiment (MIAPE) [73] and the proteomics community in ELIXIR [74] made a start on standardized documentation for proteomics experiments but is sparse on the documentation of chemometrical and statistical decisions [75]. We therefore propose to include guidelines based on our recommendations in future revisions by these communities to advance the transition from biomarker discovery into clinical application.

## Figures and Tables

**Table 1:** Confusion matrix for binary classification. The positive and negative class could be disease and control or two different types of diseases, etc.

		Actual Class	
		Positive	Negative
**Classified as**	**Positive**	True Positive (TP)	False Positive (FP)
	**Negative**	False Negative (FN)	True Negative (TN)

**Table 2:** Performance measures for binary classification based on the notation in Table 1.

Performance Measure	Formula
Number of misclassifications (NMC)	FP+FN
Accuracy	$\frac{TP+TN}{TP+FN+FP+TN}$
Sensitivity	$\frac{TP}{TP+FN}$
Specificity	$\frac{TN}{FP+TN}$
Area under the receiver operator curve (AUC)	$\frac{1}{2}\left(\frac{TP}{TP+FN}+\frac{TN}{FP+TN}\right)$
